# Biological and metabolic effects of IACS-010759, an OxPhos inhibitor, on chronic lymphocytic leukemia cells

**DOI:** 10.18632/oncotarget.25166

**Published:** 2018-05-18

**Authors:** Hima V. Vangapandu, Brandon Alston, Joshua Morse, Mary L. Ayres, William G. Wierda, Michael J. Keating, Joseph R. Marszalek, Varsha Gandhi

**Affiliations:** ^1^ Department of Experimental Therapeutics, The University of Texas MD Anderson Cancer Center, Houston, Texas, USA; ^2^ Department of Leukemia, The University of Texas MD Anderson Cancer Center, Houston, Texas, USA; ^3^ Institute of Applied Cancer Science and the Center for Co-Clinical Trials, The University of Texas MD Anderson Cancer Center, Houston, Texas, USA

**Keywords:** mitochondria, IACS-010759, metabolism, 2-dG, OxPhos

## Abstract

Blood cells from patients with chronic lymphocytic leukemia (CLL) are replicationally quiescent but transcriptionally, translationally, and metabolically active. Recently, we demonstrated that oxidative phosphorylation (OxPhos) is a predominant pathway in CLL for energy production and is further augmented in the presence of the stromal microenvironment. Importantly, CLL cells from patients with poor prognostic markers showed increased OxPhos. From these data, we theorized that OxPhos can be targeted to treat CLL. IACS-010759, currently in clinical development, is a small-molecule, orally bioavailable OxPhos inhibitor that targets mitochondrial complex I. Treatment of primary CLL cells with IACS-010759 greatly inhibited OxPhos but caused only minor cell death at 24 and 48 h. In the presence of stroma, the drug successfully inhibited OxPhos and diminished intracellular ribonucleotide pools. However, glycolysis and glucose uptake were induced as compensatory mechanisms. To mitigate the upregulated glycolytic flux, we used 2-deoxy-D-glucose in combination with IACS-010759. This combination reduced both OxPhos and glycolysis and induced cell death. Consistent with these data, low-glucose culture conditions sensitized CLL cells to IACS-010759. Collectively, these data suggest that CLL cells adapt to use a different metabolic pathway when OxPhos is inhibited and that targeting both OxPhos and glycolysis pathways is necessary for biological effect.

## INTRODUCTION

Chronic lymphocytic leukemia (CLL) is a disease of mature B cells that are CD5 positive and CD19 positive [1–3]. These cells accumulate as they fail to undergo apoptosis. CLL represents 5% of all leukemias in the Western hemisphere. CLL cells from the peripheral blood are replicationally quiescent but transcriptionally, translationally, and metabolically active [4, 5].

Several studies in solid and liquid tumors have suggested that cancer cells normally depend on both glycolysis and oxidative phosphorylation (OxPhos) [6] and that these cells acquire plasticity to adapt to metabolic conditions [7]. These processes provide cellular bioenergy as well as precursors for production of macromolecules, which are needed to maintain the high growth rate of these cells. Previous work from our laboratory has demonstrated that of the two ATP-generating pathways—glycolysis and OxPhos—the latter is associated with disease aggressiveness [8]. Furthermore, oxygen consumption rate (OCR) (a measure of OxPhos) of CLL cells varied among patient samples, ECAR (a measure of glycolysis) was relatively similar among samples. The diversity in OCR in CLL primary lymphocytes is related to the prognostic factors of the disease. CLL cells from patients with poor prognostic factors such as high Rai stage, positive ZAP-70, and unmutated IGHV had significantly higher OCRs than their counterparts with good prognoses [8]. Such differentials provide a rationale to target OxPhos therapeutically in CLL patients with relatively poor prognostic outcomes.

In addition to inherent prognostic markers, microenvironment factors have been shown to impact the sensitivity of CLL cells to chemoimmunotherapy and/or targeted therapies. Interaction of CLL primary cells with bone marrow-derived stromal cell lines has been shown to provide a survival advantage [9, 10]. In parallel with this survival benefit was an increase in the rate of gene transcription, protein translation, and metabolism in CLL cells [4, 5, 11]. Importantly, there is a significant and selective increase in OxPhos but not glycolysis in a stromal microenvironment [12]. The exclusive increase in the OCR by CLL lymphocytes provides further rationale to target OxPhos for this indolent leukemia.

OxPhos is an energy-efficient way of synthesizing ATP in the mitochondria. The mitochondrial electron transport chain consists of five complexes: NADH dehydrogenase (complex I), succinate dehydrogenase (complex II), cytochrome C reductase (complex III), cytochrome C oxidase (complex IV), and ATP synthase (complex V). Mitochondrial respiration is coupled to ATP production, in which oxygen is consumed [13]. Inhibitors of the electron transport chain impede OxPhos and curb the ATP supply.

Because CLL cell metabolism appears to rely substantially on the OxPhos pathway [8], which is further augmented and perturbed by a stromal microenvironment [12], we hypothesized that CLL cells would be selectively vulnerable to OxPhos inhibition. While there have been some attempts to design and develop OxPhos inhibitors, these did not possess qualities to become clinical candidates [14]. IACS-010759, a drug discovered by the Institute for Applied Cancer Science (IACS) at The University of Texas MD Anderson Cancer Center, is an OxPhos pathway inhibitor [15]. IACS-010759 was identified as a complex I inhibitor; at low, nanomolar levels, this small-molecule drug inhibited ATP production in isolated mitochondria [16]. The metabolic effect of IACS-010759 was apparent when malate and glutamate were used but not when succinate was supplied. Several additional genetic and pharmacologic investigations further established complex I as the target of this novel agent [16]. Additionally, this agent has clinically favorable properties including oral bioavailability, pharmacokinetic profile, and lack of adverse events [15]. Importantly, IACS-010759 is now being evaluated in a clinical trial (NCT02882321; ClinicalTrials.Gov) to test its efficacy for patients with acute myelogenous leukemia.

In the present study, we evaluated the effects of IACS-010759 in freshly isolated primary CLL cells from peripheral blood. Our investigations suggested that while there was a significant decrease in OxPhos in primary CLL cells, the biologic effect of the drug was very modest owing to a concomitant increase in the glycolysis pathway, which was measured using the extracellular acidification rate (ECAR). While all four ribonucleotide pools were reduced significantly, there was minimal cell death due to this inhibitor. Consistent with these data, simultaneous inhibition of glycolysis by either 2-deoxy-D-glucose (2-dG) or low-glucose culture conditions had a more pronounced biologic effect than the inhibitor alone did. These observations clearly suggest that CLL cells have plasticity and that when OxPhos is inhibited, the cells adapt to use a compensatory glycolysis pathway; therefore, targeting both pathways is essential for this treatment approach to have a therapeutic impact.

## RESULTS

### IACS-010759 causes minimal cell death in CLL cells

**Figure 1:**
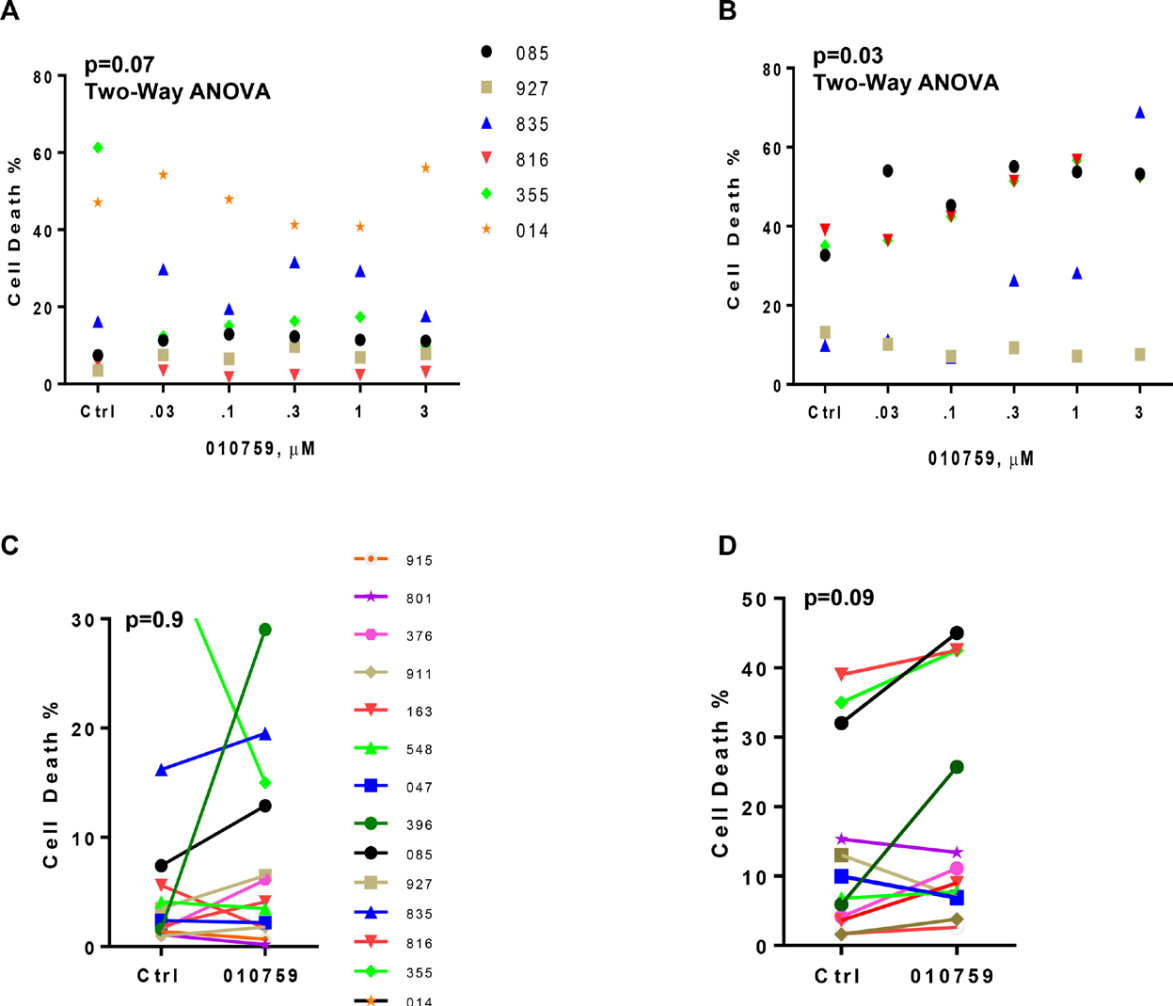

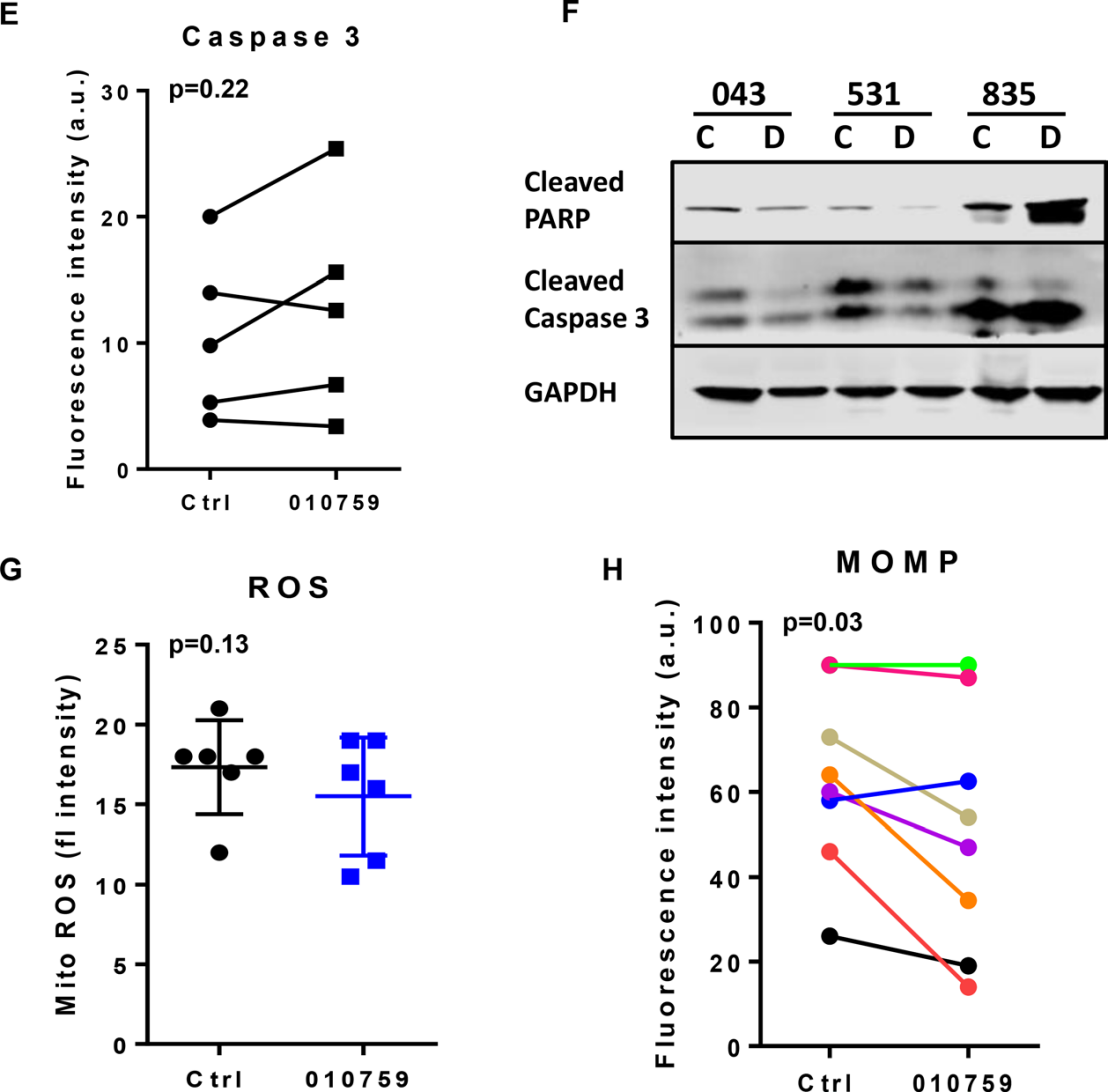
Effect of IACS-010759 on cell survival and mitochondrial functionalities of malignant CLL B cells (**A**–**B**) Cell death in primary CLL cells treated with different doses of IACS-010759 in six patient samples at 24 h (A) and 48 h (B). (**C**–**D**) Cell death in primary CLL cells incubated with or with 100 nM IACS-010759 (*n* = 14) at 24 h (C) and (*n* = 13) at 48 h (D). (**E**) Activation of caspase 3 measured by a flow cytometric assay. CLL cells that were untreated or treated with IACS-010759 (*n* = 5) were assayed for caspase 3 activity. (**F**) Immunoblot showing cleaved PARP and cleaved caspase 3 proteins in untreated or treated cells. C; Control untreated; D, drug IACS-010759-treated. Glyceraldehyde 3-phosphate dehydrogenase (GAPDH) protein was used as loading control. (**G**) CLL cells that were untreated or treated (*n* = 6) were assayed for mitochondrial ROS (mito ROS) at 24 h. (**H**) CLL cells that were untreated or treated (*n* = 8) were assayed for mitochondrial outer membrane potential (MOMP). Ctrl, untreated control; 010759, IACS-010759; ANOVA, analysis of variance; a.u. absorbance unit.

Five concentrations (30 nM, 100 nM, 300 nM, 1 μM, and 3 μM) of IACS-010759 were tested on six patient samples with incubation times of 24 and 48 h (Figure 1A, 1B). In untreated time-matched samples (control), cell death rates ranged from 5% to 60%. After treatment with the drug, these values increased moderately at both 24 and 48 h (Figure 1A); cell death was limited at both time points. A concentration of 100 nM was used for all experiments thereafter. At this dose, additional samples were tested for cell death at 24 and 48 h (Figure 1C, 1D). Even in these samples, compared to time-matched control (DMSO only incubations) samples, the cell death was minimal in IACS-010759-treated samples. Caspase 3 levels were measured by flow cytometry. Caspase 3 was not induced in treated samples compared with untreated time-matched counterparts (Figure 1E). Only one of three CLL samples showed an increase in cleaved poly(ADP-ribose) polymerase levels (Figure 1F). Since cell death was very minimal with IACS-010759, we tested for autophagy induction upon drug treatment. CLL cells were treated with IACS-010759 for 24 or 48 h and were then stained with acridine orange and analyzed by flow cytometry. A representative flow panel of one patient sample is shown in Supplementary Figure 1. Compared to time-matched untreated (Control) samples, data in several samples suggested no significant induction of autophagy (five samples -24 h; six samples - 48h).

Mitochondrial ROS level was measured in treated samples by flow cytometry (Figure 1G) and no significant change in ROS was observed in six patient samples after 24 h of incubation with the drug. Similarly, mitochondrial outer membrane potential was measured in eight samples after incubation with 100 nM IACS-010759 for 24 h (Figure 1H). Again, not much change was observed for this parameter.

### IACS-010759 inhibits OCR and increases glycolysis in CLL cells

**Figure 2:**
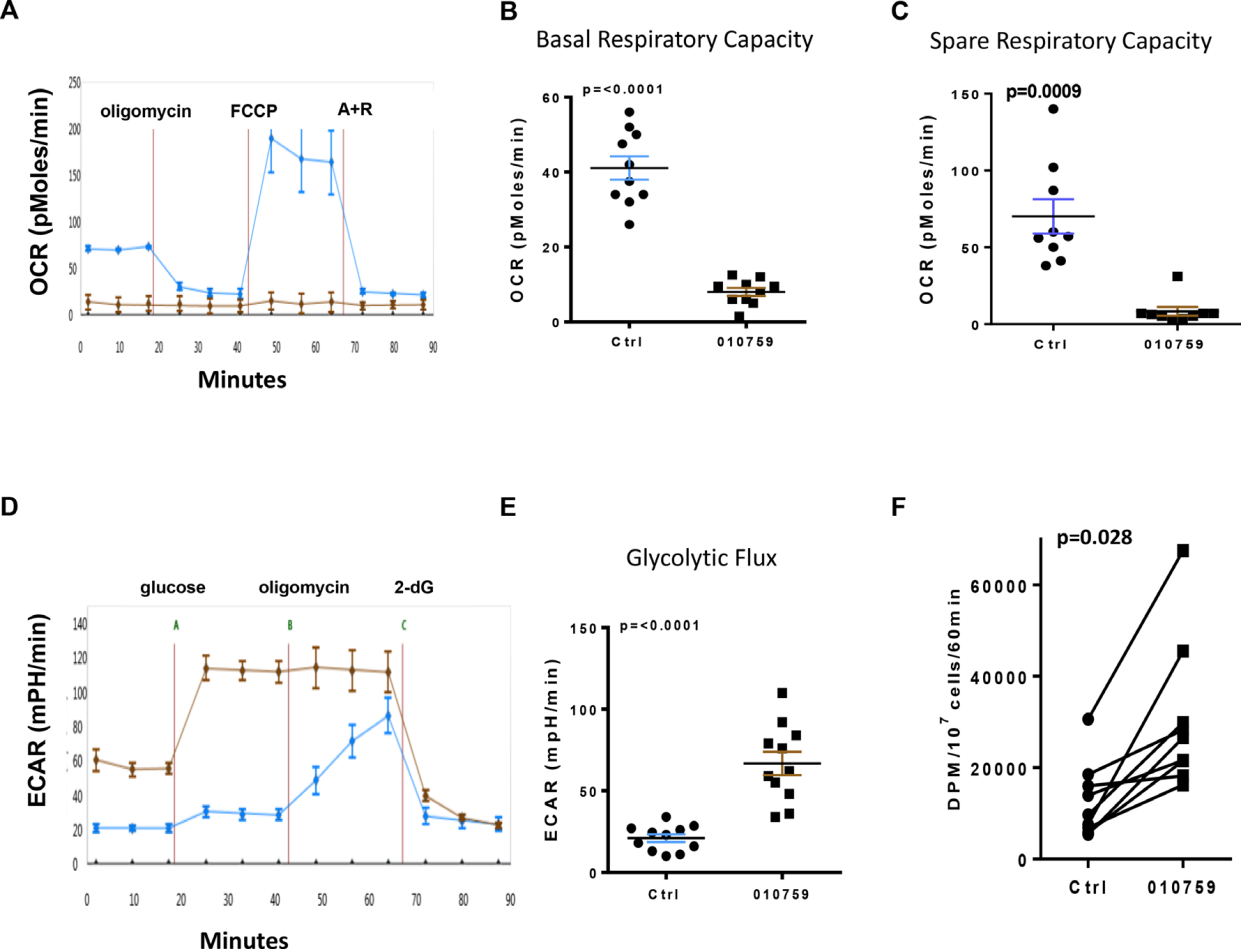
Impact of IACS-010759 on mitochondrial OxPhos and glycolysis in CLL cells CLL cells were untreated or were treated with 100 nM IACS-010759. Equal numbers of untreated and IACS-010759-treated CLL cells (100 nM) were plated for the XF assay. Five technical replicates were used for OCR and ECAR assays. (**A**) XF cell mitochondrial stress test profile of a CLL sample. CLL cells that were untreated (blue curve), or treated with IACS-010759 (brown curve) were used for the assay. (**B**) Basal OCR of untreated (blue line) and treated (brown line) CLL cells were analyzed for OxPhos (*n* = 10). (**C**) Changes in spare respiratory capacity of untreated (blue line) and treated (brown line) CLL cells. (**D**) XF glycolysis stress test profile of the CLL samples analyzed for OxPhos in A. (**E**) Glycolytic flux of untreated and treated CLL cells that were analyzed for OxPhos (*n* = 11). (**F**) Changes in glucose uptake in CLL cells upon treatment. Untreated and treated CLL cells were assessed for [^3^H]-deoxy-d-glucose uptake (*n* = 9). Ctrl, untreated control; 010759, IACS-010759. FCCP, carbonylcyanide-4-trifluoromethoxyphenylhydrazone; A+R, antimycin and rotenone; DPM, disintegration per minute.

CLL cells were incubated with 100 nM IACS-010759 for 24 h and later assayed for changes in mitochondrial OCR and ECAR. Untreated cells showed the expected increase in spare respiratory capacity upon addition of uncoupler carbonylcyanide-4-trifluoromethoxyphenylhydrazone (FCCP). In drug-treated cells, basal OCR was greatly inhibited followed by a drastic decrease in spare respiratory capacity (after addition of FCCP) compared with the untreated control (Figure 2A). Similar assays were done in 10 patient samples where basal respiratory capacity (Figure 2B) and spare respiratory capacity showed a similar trend after incubation with the drug (Figure 2C). Glycolysis was measured simultaneously in these patient samples. An increase in glycolytic flux was observed in treated cells compared with untreated cells (Figure 2D). A similar increase in glycolytic flux was noted when an additional 11 samples were evaluated (Figure 2E). Because glycolytic flux increased, we measured glucose consumption by the cells (substrate for glycolysis). 2-dG was used to measure glucose uptake in untreated and after a 24 h treatment with IACS-010759 (Figure 2F). Glucose uptake was significantly increased after treatment in nine samples.

### IACS-010759 decreases intracellular ribonucleotide triphosphate pools in CLL

**Figure 3:**
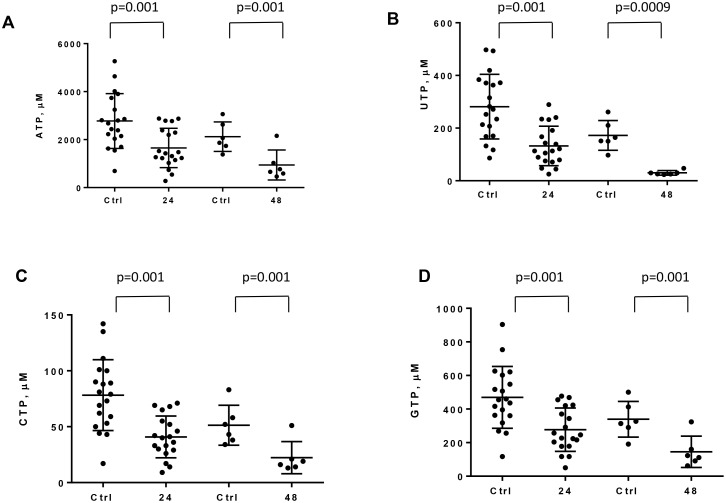
Effect of IACS-010759 on ribonucleotide levels in CLL cells CLL cells were untreated or were treated with 100 nM IACS-010759 for 24 or 48 h. High-pressure liquid chromatography was performed to assess the levels of intracellular ribonucleotides. All four NTP pools—ATP (**A**), UTP (**B**), CTP (**C**), and GTP (**D**)—were measured after 24 h (*n* = 19) or 48 h of IACS-010759 (*n* = 6) compared with controls. Ctrl, untreated control; 24, 24-h treatment with IACS-010759; 48, 48-h treatment with IACS-010759.

CLL cells were incubated with 100 nM IACS-010759 for 24 h (*n* = 19) and 48 h (*n* = 6). Figure 3 shows that the ribonucleotide pools decreased at both time points upon drug treatment. ATP concentrations in CLL cells from these patients ranged from 693 to 5267 μM with a mean of 2775 μM. This mean value decreased to 1652 μM after 24 h. At 48 h, the levels further decreased from 2124 to 943 μM.

### Impact of IACS-010759 on CLL cells in presence of stroma

**Figure 4:**
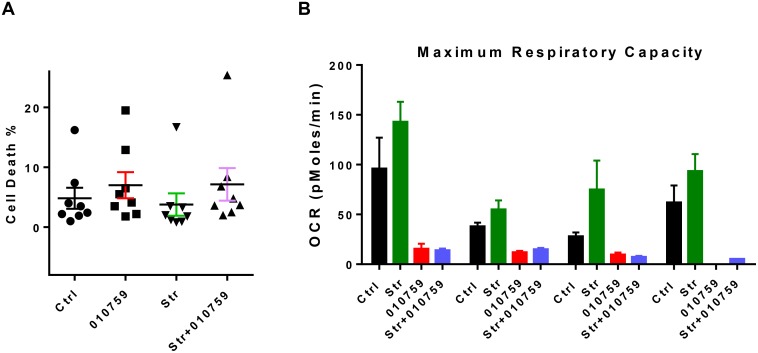
Impact of IACS-010759 on CLL cells co-cultured on stroma (**A**) Effect of IACS-010759 on CLL cells cultured in suspension or on stroma. CLL cells were untreated in suspension, treated with IACS-010759 in suspension, untreated on stroma, or treated with IACS-010759 on stroma. Cell death was measured as frequency of annexin V/propidium iodide-positive cells. Cells were obtained from the peripheral blood of patients with CLL (*n* = 8). The one way ANOVA p value is 0.0425. (**B**) Measurement of maximum respiratory capacity as OCR in four CLL samples tested either in suspension or after stromal co-culture and untreated or treated with IACS-010759 for 24 h. The p values are, C vs 010759, *p* ≤ 0.015; 010759 vs stroma, *p* = 0.08, Ctrl, untreated control; 010759, 100 nM IACS-010759; Str, stromal co-culture.

CLL cells were cultured in suspension or in the presence of stroma with or without 100 nM IACS-010759. There was some CLL cell death observed in untreated time-matched control (Ctrl) samples. Incubation with IACS-010759 did not increase cell death. There was some protection of cell death when CLL lymphocytes were co-cultured with stroma. The drug resulted in minor increase in cell death when CLL lymphocytes were cultured in the presence of stroma (Figure 4A). Mitochondrial stress tests were performed to analyze the impact of the drug on OCR in CLL cells in presence of the stromal cells. OCR was augmented when CLL cells were cultured on stroma cells (Figure 4B). IACS-010759 mitigated the OCR induced by stroma cells, bringing the OCR levels in CLL co-cultured with stroma to the same level as that seen in suspension cultures with the drug.

### Effect of IACS-010759 in combination with glycolysis inhibitor 2-dG

**Figure 5:**
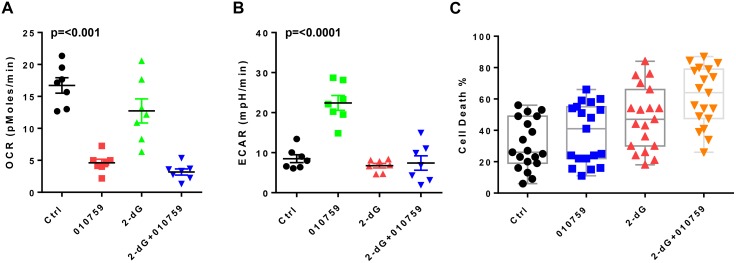

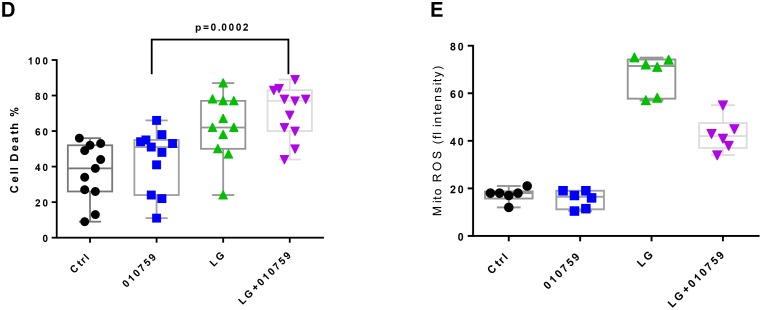
Effect of combination of IACS-010759 and 2-dG or low-glucose conditions on CLL cells (**A**) Basal OCR was measured in CLL cells (*n* = 7) that were untreated or treated with 100 nM IACS-010759 alone, 5 mM 2-dG alone, or a combination of IACS-010759 and 2-dG. The p values are, Ctrl vs 010759, *p ≤* 0.0001; 010759 vs 2dG, *p* = 0.0054; 010759 vs combo, *p* = 0.07; 2dG vs combo, *p* = 0.0017. (**B**) Effect of single agent versus combination on ECAR. Measurement of ECAR in same seven patient samples as in A. The p values are Ctrl vs 010759, *p* = 0.0003; 010759 vs 2dG, *p* ≤ 0.0001; 010759 vs combo *p ≤* 0.0001; 2dG vs combo, *p* = 0.69. (**C**) Impact of single agent and combination on cell death in CLL cells obtained from patients (*n* = 19). Induction of apoptosis was measured using annexin V/propidium iodide staining. The p values are Ctrl vs 010759, *p* = 0.011; 010759 vs 2dG, *p* = 0.011; 010759 vs combo, *p* ≤ 0.0001; 2dG vs combo, *p* = 0.0005. (**D**) Effect of low glucose conditions (5 mM) on CLL cell death and impact of addition of IACS-010759 during low glucose culture conditions. CLL cells from patients (*n* = 11) were either untreated, treated with IACS-010759, low glucose medium, and low glucose medium with IACS-010759 for 24 h and apoptosis was measured using annexin V/propidium iodide staining. The p values are Ctrl vs 010759, *p* = 0.011; 010759 vs LG, *p* = 0.0011; 010759 vs combo, *p* = 0.0002; LG vs combo, *p* = 0.1. (**E**) Changes in mitochondrial ROS (mito ROS) in CLL cells. CLL cells were treated as in D, and mito ROS was measured as described in the Methods section. The p values are Ctrl vs 010759, *p* = 0.13; Ctrl vs LG, *p* = 0.0001; 010759 vs combo, *p* = 0.001; LG vs combo, *p* = 0.0029. Ctrl, untreated control; 010759, 100 nM IACS-010759; LG, low-glucose culture conditions; fl, fluorescent.

Because glycolysis was upregulated upon IACS-010759 treatment of CLL cells, we next tested a combination of an OxPhos inhibitor and a glycolysis inhibitor. CLL cells were cultured in suspension for 24 h in each of the following conditions: untreated, with 100 nM IACS-010759, with 5 mM 2-dG, and with IACS-010759 combined with 2-dG. In cells incubated with 2-dG, the OCR decreased slightly compared with the controls (Figure 5A). In cells incubated with the combination, the OCR was further and significantly reduced compared with controls and even compared with cells treated with IACS-010759 alone. When the cells were assayed for glycolysis (ECAR) under these culture conditions, glycolysis increased, as expected, in the cells incubated with IACS-010759 compared with controls; but in cells incubated with the combination, ECAR significantly decreased and reached almost basal levels (Figure 5B).

Apoptosis was also measured under these culture conditions. Compared to time-matched untreated control CLL samples (Ctrl), IACS-010759 caused minimal cell death, but 2-dG treatment resulted in noticeably more cell death, and the combination resulted in higher cell death than either individual drug treatment (Figure 5C). Another method of blocking glycolysis is reducing the glucose concentration in the media. We tested low-glucose conditions (5 mM final concentration) separately from and in combination with IACS-010759 and quantitated the resulting apoptosis levels. As seen with 2-dG, low-glucose conditions caused significantly higher cell death rates compared with no treatment, and IACS-010759 in low-glucose conditions further increased cell death (Figure 5D). Mitochondrial ROS was also measured under these culture conditions. Cells in low-glucose cultures showed a significant increase in ROS levels compared with controls (Figure 5E). The addition of IACS-010759 to low-glucose cultures decreased the ROS levels; however, these ROS levels were higher than those of untreated cells or cells treated with IACS-010759 alone.

### Impact of IACS-010759 on healthy donor PBMCs and B cells

**Figure 6:**
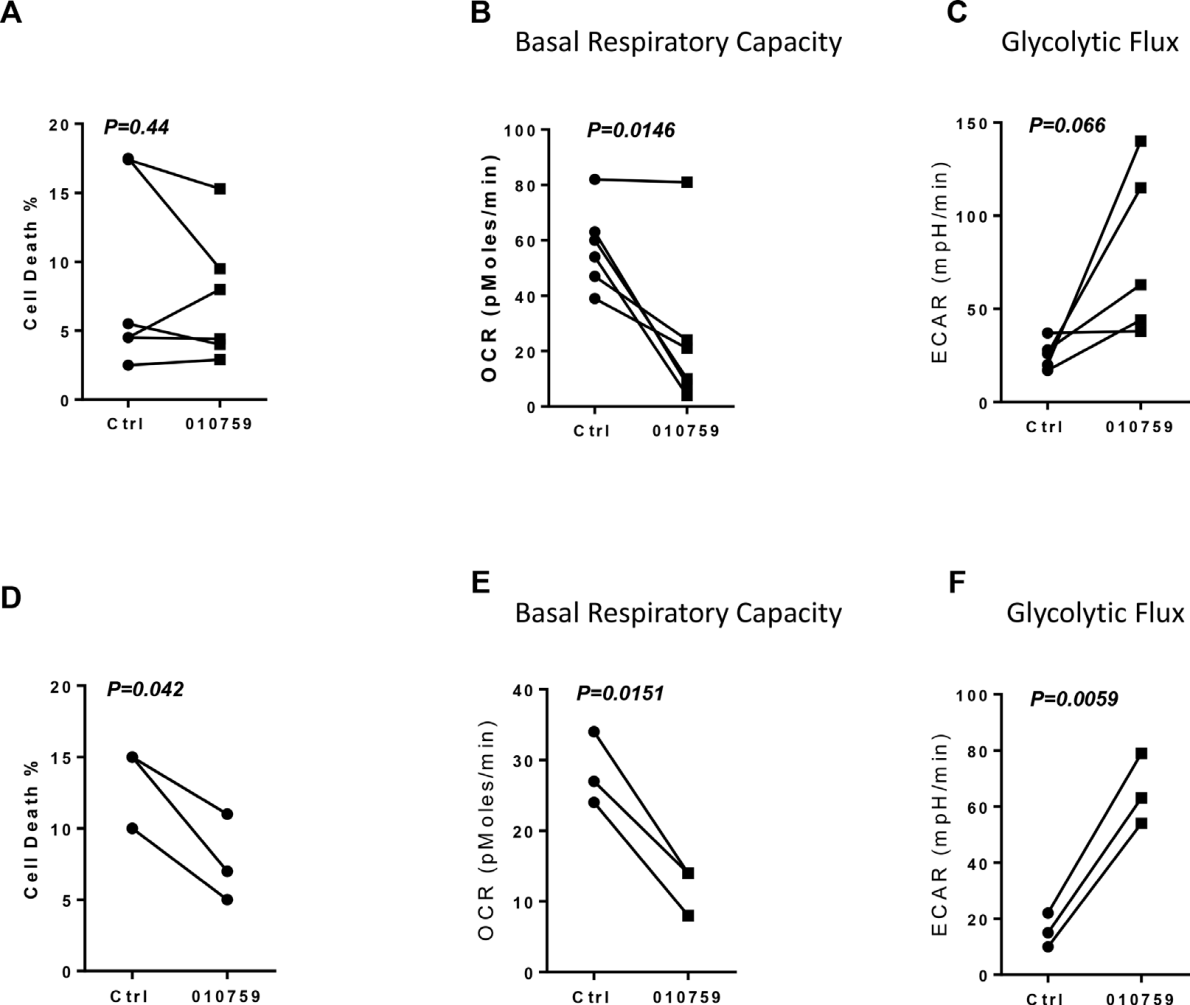
Effect of IACS-010759 on healthy donor PBMCs or B cells (**A**) Cell death rate in healthy donor PBMCs that were untreated or were treated with 100 nM IACS-010759 (*n* = 6). (**B**) Changes in basal respiratory capacity measured as OCR in PBMCs upon IACS-010759 incubation for 24 h followed by an XF assay. (**C**) ECAR in five healthy donor PBMCs samples that were untreated or treated for 24 h. (**D**) Cell death rate in untreated or treated healthy donor B cells (*n* = 3). (**E**) Measurement of OCR in healthy donor B cells (*n* = 3) before and after treatment with IACS-010759. (**F**) ECAR measurement in healthy donor B cells before and after IACS-010750 treatment. Ctrl, untreated control; 010759, 100 nM IACS-010759 for 24 h.

We assessed the effect of IACS-010759 on PBMCs isolated from peripheral blood obtained from six healthy donors. Again, cytotoxicity was limited (Figure 6A). The PBMCs were also assayed for OCR and ECAR. The results seen with the CLL cells were recapitulated with the healthy donor PBMCs. The OCR was mitigated in five of six donors (Figure 6B), while ECAR was increased in four of the five donors tested (Figure 6C). Finally, healthy donor B cells were cultured and incubated with IACS-010759. Time-matched controls had 10%-15% cell death rates, and these rates were decreased with IACS-010759 rather than increased (Figure 6D). As seen with the healthy donor PBMCs, basal OCR was decreased and ECAR increased in B cells from three healthy donors upon incubation with IACS-010759 (Figures 6E and 6F).

## DISCUSSION

ATP is the molecular currency of the cell, and this cellular bioenergy is generated through two major pathways: mitochondrial OxPhos and cytoplasmic glycolysis. In normal cells, OxPhos is the predominant pathway to generate ATP [17]. Under anaerobic conditions, glycolysis is utilized to produce ATP. In cancer cells, the glycolysis pathway is upregulated, followed by lactic acid fermentation in the cytosol which is known as the Warburg effect [18]. In contrast, in normal cells, glycolysis is followed by oxidation of pyruvate using the OxPhos pathway of mitochondrial machinery. Furthermore, mitochondria in cancer cells are reported to be dysfunctional, although it is not known if they impact ATP production [19]. On the other hand, mitochondrial DNA and biogenesis are associated with not only respiration and tumorigenesis but also oxidative phosphorylation and metastasis [20–22].

Glycolysis occurs in the cytoplasm. Glucose is the substrate, which through a series of ten-enzyme-catalyzed reactions is converted to pyruvate. This pyruvate in turn is converted to lactic acid, the end product of glycolysis. In cancer cells, glucose is converted to pyruvate irrespective of oxygen conditions and yields 4 mol of ATP per 1 mol of glucose [23]. ATP is directly formed through phosphate transfer from substrate to ATP, or substrate phosphorylation. Some of the pyruvate enters the tricarboxylic cycle, whereas most of the end product, lactic acid, is flushed out of the cell.

OxPhos occurs in the mitochondria of cells. Glutamine, glucose, or fatty acids are the NADH suppliers for the electron transport chain. ATP is formed through a series of redox reactions involving oxygen as the final electron acceptor. The series of oxidative reduction reactions occur through the four complexes of the electron transport chain, which then generates an electrochemical gradient in the inner mitochondrial membrane [24]. Protons return to the mitochondrial matrix through ATP synthase, and this process is coupled to ATP synthesis. A total of 36 mol of ATP are produced per 1 mol of glucose [23].

There have been several attempts to identify the metabolic program of tumor tissue and cancer cell survival and proliferation; however, it is not completely clear which process (OxPhos or glycolysis) is preferred. Although OxPhos is a more efficient pathway for energy production, it may not be the preferred pathway in cancer cells. Mitochondrial DNA mutations are common in neoplastic cells and hence may not have functionally active electron transport chains [25], although this relationship is not completely established [26]. For solid tumor cells in general, cells are highly proliferative as well as metabolically active; however, they are generally in hypoxic conditions. Nonetheless, these cells are highly plastic and adapt to this anaerobic condition by upregulating glycolysis and making it a primary route for production of cellular bioenergy [18]. In contrast to this common view of metabolic switch, newer studies suggest that both glycolysis and oxidative phosphorylation are enhanced in tumor tissue compared with surrounding cells [27]. Irrespective of the preferred metabolic program and associated controversies, it is becoming clear that tumor cells are highly plastic and adapt to conditions such as low oxygen and or low nutrients by reprogramming their metabolism.

Peripheral blood CLL cells, in contrast to highly proliferative solid tumor cells, are replicationally quiescent. Compared to proliferative malignant B-cell lines, that show high glycolysis rate (measured as ECAR), CLL cells showed low ECAR. OxPhos, on the other hand varied and correlated with disease aggressiveness [8]. Reliance on OxPhos for production of cellular bioenergy was reported in quiescent melanoma cells and leukemia stem cells [28, 29]. Increased aggressiveness of this tumor subtype is associated with increased OxPhos; for example, CLL lymphocytes from individuals with advanced Rai stage, ZAP-70-positive disease, or disease with unmutated IGHV show increased OxPhos in contrast to ZAP-70-negative or IGHV-mutated subtypes [8] Microenvironment further aided OxPhos induction. Although OxPhos was the preferred route of energy production in CLL cells from peripheral blood in all these conditions, challenging these cells with IACS-010759 clearly demonstrated that the cells adapt to this adverse condition by switching to glycolysis (Figure 2B). This plasticity provided a survival advantage to the CLL cells, even under potent inhibition of OxPhos. Such an adaptation in cancer cells has been seen with other OxPhos inhibitors. Arctigenin, when used for treatment of advanced pancreatic cancers, selectively killed OxPhos-dependent cells [30]. With IACS-010759, KRAS-negative pancreatic cancer cells were selectively targeted since their glycolysis remained low, while KRAS-positive cells adapted by upregulating glycolysis as a compensatory mechanism [14]. Atovaquone acts as a selective OxPhos inhibitor by targeting the coenzyme Q10 dependence of mitochondrial complex III. When atovaquone was used in MCF7 breast cancer cells, it inhibited OxPhos but also induced glycolysis [31], as seen with IACS-010759 in the present study. In general, these data suggest that when challenged, the cancer cells amend their metabolic profile.

The plasticity of CLL cells has also been observed in the presence of stromal microenvironment. While short-term interaction (24 h) with a variety of stromal cell lines have been found to significantly increase OCR and not glycolysis, longer incubations (6 days) with bone marrow stromal cell lines propelled glycolysis [32]. Our findings are in concert with these observations and suggest treatment-induced adaptation and plasticity of CLL cells.

Several agents are being tested to target OxPhos [33]. Because IACS-010759 induces minimal cell death in CLL, probably because of the compensatory increase in glycolysis, we tested inhibition of both pathways as a therapeutic strategy. We used two approaches: the use of 2-dG to mitigate glycolysis and the use of low-glucose conditions to reduce glycolytic flux. Some cell death was seen with 2-dG itself, but its combination with IACS-010759 led to higher cell death rates (Figure 5C). A previous study showed that 2-dG impacted the cell viability of lymphoma cells as a single agent and reduced cell numbers synergistically with PI3K inhibitors and c-MYC antagonists [34]. Also, inhibition of glycolysis by 2-dG in ovarian cancer cell lines reduced cell proliferation [35]. Similarly, 2-dG as a single agent was used to selectively kill acute lymphoblastic leukemia cells [36]. Consistent with our observations, combination of 2-dG with electron transport chain blocker antimycin A or rotenone significantly increased cell death compared with each drug alone [37].

In a Tcl-1 adoptive transfer CLL mouse model system, single-agent IACS-010759 induced little biologic effect and minimal adverse events (data not shown). The same system needs to be used to test a combination of both IACS-010759 and a glycolysis inhibitor to validate our current data in the *in vitro* model system. In conclusion, our data suggest that CLL cells adapt to use a different metabolic pathway when OxPhos is inhibited and that targeting both OxPhos and glycolysis pathways is necessary for this approach to have therapeutic benefit.

## MATERIALS AND METHODS

### Patient and healthy donor blood collection

Peripheral blood samples were collected in green-top tubes from 43 CLL patients (Supplementary Table 1). All patients gave written informed consent in accordance with the Declaration of Helsinki and under a protocol approved by the Institutional Review Board of MD Anderson Cancer Center. Healthy donor blood was obtained from Gulf Coast Regional Blood Center in Houston, Texas.

### Drugs

IACS-010759 was obtained from the Institute for Applied Cancer Science at MD Anderson. 2-dG was obtained from Dr. Waldemar Priebe's group at MD Anderson. IACS-010759 was dissolved in dimethyl sulfoxide. 2-dG was dissolved in distilled H_2_O.

### Cell isolation

Peripheral blood mononuclear cells (PBMCs) from blood samples of CLL patients or healthy donors were separated by Ficoll-Hypaque density centrifugation (Atlanta Biologicals, Flowery Branch, GA, USA). Cells were cultured in Roswell Park Memorial Institute 1640 (RPMI-1640) medium with L-glutamine and 10% human serum. All experiments were performed using freshly isolated CLL cells; the purity of this cell population was ≥95%. Viability of these cells right after isolation is between 95–100%; for each cell death experiment, time-matched control (ctrl) was used to determine the viability after 24 or 48 hours of incubation in medium.

### CLL stromal cell co-cultures

PBMCs from blood samples of CLL patients were co-cultured with stromal cells (NK.Tert) at a ratio of 100 CLL cells to 1 stromal cell. Generally, the cultures were maintained for 24 h. The culture methods and optimization are described in detail in prior publications [38].

### Cytotoxicity assays

To identify and quantitate apoptotic and necrotic cells, we stained CLL cells with annexin V and propidium iodide and counted the cells using flow cytometry as described previously [39].

### Extracellular flux assays

Extracellular flux assays (Seahorse Bioscience, North Billerica, MA, USA) were used to measure the OCR and ECAR of CLL cells. CLL cells (5 × 10^5^) were plated in RPMI-1640 and 10% human serum in six-well plates with or without IACS-010759 for 24 h. Cells were counted and plated onto XF microplates. RPMI-1640 medium was replaced with XF base (to measure OCR) or glycolysis base (to measure ECAR) media as recommended by Seahorse Bioscience. Five technical replicates for each condition were plated [8].

### Mitochondrial reactive oxygen species and membrane potential

CLL cells were incubated with either dimethyl sulfoxide (control) or IACS-010759 (100 nM) for 24 h. A total of 10^6^ cells were stained with MitoSOX Red and tetramethylrhodamine ethyl ester perchlorate and were analyzed using flow cytometry for mitochondrial reactive oxygen species (ROS) and mitochondrial outer membrane potential, respectively.

### Caspase 3 assay

CLL cells were incubated with either dimethyl sulfoxide (control) or IACS-010759 (100 nM) for 24 h. A total of 10^6^ cells were stained with CellEvent Caspase-3/7 Green Detection Reagent (Thermo Fisher Scientific, Waltham, MA, USA) and analyzed using flow cytometry to detect activated caspase 3.

### Ribonucleotide pool measurement

CLL cells were incubated with or without IACS-010759, and ribonucleotides were extracted from cells using the perchloric acid method. Extracts were neutralized and used for high-pressure liquid chromatography. Pools were separated using an anion exchange column and high-pressure liquid chromatography as described previously [40]. Standard ribonucleotides were used to create a standard curve, which was used to quantitate nucleotide pools; and the concentration was determined on the basis of mean cell volume. Mean cell volumes were determined by a Coulter Counter (Beckman Coulter, Indianapolis, IN, USA).

### Glucose uptake assay

CLL cells were incubated with either dimethyl sulfoxide (control) or IACS-010759 (100 nM) for 24 h. CLL cells were then washed with 1X phosphate-buffered saline twice before resuspension in glucose-free media. [^3^H] 2-dG (0.5 μL, specific activity 28 Ci/mmol) was added (both reagents from PerkinElmer) to the cells and incubated for 1 h. The cells were washed with ice-cold 1X phosphate-buffered saline twice and quenched with 1N NaOH. Radioactivity was quantified using the scintillation counter.

### Autophagy

A total of 10^6^ CLL cells were either untreated or treated with IACS-010759 for 24 or 48 h, washed and stained with 1 mL of Acridine Orange (10 μg/mL) (Invitrogen, Carlsbad, CA, USA) and analyzed using flow cytometry for autophagy. Changes in acridine orange positive population were determined.

### Statistical analysis

Student *t*-tests (two-tailed) and analysis of variance were performed using Prism 6 software (GraphPad Software, La Jolla, CA, USA) with a significance level of < 0.05.

## SUPPLEMENTARY MATERIALS FIGURES AND TABLES





## References

[1] Chiorazzi N, Rai KR, Ferrarini M (2005). Chronic lymphocytic leukemia. N Engl J Med.

[2] Ghamlouch H, Nguyen-Khac F, Bernard OA (2017). Chronic lymphocytic leukaemia genomics and the precision medicine era. Br J Haematol.

[3] Guieze R, Wu CJ (2015). Genomic and epigenomic heterogeneity in chronic lymphocytic leukemia. Blood.

[4] Balakrishnan K, Burger JA, Fu M, Doifode T, Wierda WG, Gandhi V (2014). Regulation of Mcl-1 expression in context to bone marrow stromal microenvironment in chronic lymphocytic leukemia. Neoplasia.

[5] Balakrishnan K, Burger JA, Quiroga MP, Henneberg M, Ayres ML, Wierda WG, Gandhi V (2010). Influence of bone marrow stromal microenvironment on forodesine-induced responses in CLL primary cells. Blood.

[6] Zheng J (2012). Energy metabolism of cancer: Glycolysis versus oxidative phosphorylation (Review). Oncol Lett.

[7] Cairns RA, Mak TW (2017). Fire and water: Tumor cell adaptation to metabolic conditions. Exp Cell Res.

[8] Vangapandu HV, Havranek O, Ayres ML, Kaipparettu BA, Balakrishnan K, Wierda WG, Keating MJ, Davis RE, Stellrecht CM, Gandhi V (2017). B-cell Receptor Signaling Regulates Metabolism in Chronic Lymphocytic Leukemia. Mol Cancer Res.

[9] Burger JA, Tsukada N, Burger M, Zvaifler NJ, Dell’Aquila M, Kipps TJ (2000). Blood-derived nurse-like cells protect chronic lymphocytic leukemia B cells from spontaneous apoptosis through stromal cell-derived factor-1. Blood.

[10] Burger JA, Zvaifler NJ, Tsukada N, Firestein GS, Kipps TJ (2001). Fibroblast-like synoviocytes support B-cell pseudoemperipolesis via a stromal cell-derived factor-1- and CD106 (VCAM-1)-dependent mechanism. J Clin Invest.

[11] Balakrishnan K, Burger JA, Wierda WG, Gandhi V (2009). AT-101 induces apoptosis in CLL B cells and overcomes stromal cell-mediated Mcl-1 induction and drug resistance. Blood.

[12] Vangapandu HV, Ayres ML, Bristow CA, Wierda WG, Keating MJ, Balakrishnan K, Stellrecht CM, Gandhi V (2017). The Stromal Microenvironment Modulates Mitochondrial Oxidative Phosphorylation in Chronic Lymphocytic Leukemia Cells. Neoplasia.

[13] Mitchell P (1961). Coupling of phosphorylation to electron and hydrogen transfer by a chemi-osmotic type of mechanism. Nature.

[14] Viale A, Pettazzoni P, Lyssiotis CA, Ying H, Sanchez N, Marchesini M, Carugo A, Green T, Seth S, Giuliani V, Kost-Alimova M, Muller F, Colla S (2014). Oncogene ablation-resistant pancreatic cancer cells depend on mitochondrial function. Nature.

[15] Protopopova MB, Bardenhagen M, Bristow J, Caroll C, Chang C, Feng E, Gay N, Geck J, Do M, Greer M, Konopleva M, Matre P, Kang Z (2015). IACS-10759: A novel OXPHOS inhibitor which selectively kill tumors with metabolic vulnerabilities. Cancer Res.

[16] Molina JR, Sun Y, Protopopova M, Gera S, Bandi M, Bristow C, McAfoos T, Morlacchi P, Ackroyd J, Agip AN, Al-Atrash G, Asara J, Bardenhagen J (2018). An inhibitor of oxidative phosphorylation exploits cancer vulnerability. Nat Med.

[17] Pfeiffer T, Schuster S, Bonhoeffer S (2001). Cooperation and competition in the evolution of ATP-producing pathways. Science.

[18] Koppenol WH, Bounds PL, Dang CV (2011). Otto Warburg's contributions to current concepts of cancer metabolism. Nat Rev Cancer.

[19] Boland ML, Chourasia AH, Macleod KF (2013). Mitochondrial dysfunction in cancer. Front Oncol.

[20] LeBleu VS, O’Connell JT, Gonzalez Herrera KN, Wikman H, Pantel K, Haigis MC, de Carvalho FM, Damascena A, Domingos Chinen LT, Rocha RM, Asara JM, Kalluri R (2014). PGC-1alpha mediates mitochondrial biogenesis and oxidative phosphorylation in cancer cells to promote metastasis. Nat Cell Biol.

[21] Tan AS, Baty JW, Dong LF, Bezawork-Geleta A, Endaya B, Goodwin J, Bajzikova M, Kovarova J, Peterka M, Yan B, Pesdar EA, Sobol M, Filimonenko A (2015). Mitochondrial genome acquisition restores respiratory function and tumorigenic potential of cancer cells without mitochondrial DNA. Cell Metab.

[22] Viale A, Corti D, Draetta GF (2015). Tumors and mitochondrial respiration: a neglected connection. Cancer Res.

[23] Vander Heiden MG, Cantley LC, Thompson CB (2009). Understanding the Warburg effect: the metabolic requirements of cell proliferation. Science.

[24] Gautheron DC (1984). Mitochondrial oxidative phosphorylation and respiratory chain: review. J Inherit Metab Dis.

[25] Wallace DC (2012). Mitochondria and cancer. Nat Rev Cancer.

[26] Schon EA, DiMauro S, Hirano M (2012). Human mitochondrial DNA: roles of inherited and somatic mutations. Nat Rev Genet.

[27] DeBerardinis RJ, Chandel NS (2016). Fundamentals of cancer metabolism. Sci Adv.

[28] Lagadinou ED, Sach A, Callahan K, Rossi RM, Neering SJ, Minhajuddin M, Ashton JM, Pei S, Grose V, O’Dwyer KM, Liesveld JL, Brookes PS, Becker MW, Jordan CT (2013). BCL-2 inhibition targets oxidative phosphorylation and selectively eradicates quiescent human leukemia stem cells. Cell Stem Cell.

[29] Roesch A, Vultur A, Bogeski I, Wang H, Zimmermann KM, Speicher D, Korbel C, Laschke MW, Gimotty PA, Philipp SE, Krause E, Patzold S, Villanueva J (2013). Overcoming intrinsic multidrug resistance in melanoma by blocking the mitochondrial respiratory chain of slow-cycling JARID1B(high) cells. Cancer Cell.

[30] Brecht K, Riebel V, Couttet P, Paech F, Wolf A, Chibout SD, Pognan F, Krahenbuhl S, Uteng M (2017). Mechanistic insights into selective killing of OXPHOS-dependent cancer cells by arctigenin. Toxicol *In Vitro*.

[31] Fiorillo M, Lamb R, Tanowitz HB, Mutti L, Krstic-Demonacos M, Cappello AR, Martinez-Outschoorn UE, Sotgia F, Lisanti MP (2016). Repurposing atovaquone: targeting mitochondrial complex III and OXPHOS to eradicate cancer stem cells. Oncotarget.

[32] Jitschin R, Braun M, Qorraj M, Saul D, Le Blanc K, Zenz T, Mougiakakos D (2015). Stromal cell-mediated glycolytic switch in CLL cells involves Notch-c-Myc signaling. Blood.

[33] Weinberg SE, Chandel NS (2015). Targeting mitochondria metabolism for cancer therapy. Nat Chem Biol.

[34] Broecker-Preuss M, Becher-Boveleth N, Bockisch A, Duhrsen U, Muller S (2017). Regulation of glucose uptake in lymphoma cell lines by c-MYC- and PI3K-dependent signaling pathways and impact of glycolytic pathways on cell viability. J Transl Med.

[35] Sun L, Yin Y, Clark LH, Sun W, Sullivan SA, Tran AQ, Han J, Zhang L, Guo H, Madugu E, Pan T, Jackson AL, Kilgore J (2017). Dual inhibition of glycolysis and glutaminolysis as a therapeutic strategy in the treatment of ovarian cancer. Oncotarget.

[36] Gu L, Yi Z, Zhang Y, Ma Z, Zhu Y, Gao J (2017). Low dose of 2-deoxy-D-glucose kills acute lymphoblastic leukemia cells and reverses glucocorticoid resistance via N-linked glycosylation inhibition under normoxia. Oncotarget.

[37] Fath MA, Diers AR, Aykin-Burns N, Simons AL, Hua L, Spitz DR (2009). Mitochondrial electron transport chain blockers enhance 2-deoxy-D-glucose induced oxidative stress and cell killing in human colon carcinoma cells. Cancer Biol Ther.

[38] Kurtova AV, Balakrishnan K, Chen R, Ding W, Schnabl S, Quiroga MP, Sivina M, Wierda WG, Estrov Z, Keating MJ, Shehata M, Jager U, Gandhi V (2009). Diverse marrow stromal cells protect CLL cells from spontaneous and drug-induced apoptosis: development of a reliable and reproducible system to assess stromal cell adhesion-mediated drug resistance. Blood.

[39] Balakrishnan K, Peluso M, Fu M, Rosin NY, Burger JA, Wierda WG, Keating MJ, Faia K, O’Brien S, Kutok JL, Gandhi V (2015). The phosphoinositide-3-kinase (PI3K)-delta and gamma inhibitor, IPI-145 (Duvelisib), overcomes signals from the PI3K/AKT/S6 pathway and promotes apoptosis in CLL. Leukemia.

[40] Stellrecht CM, Vangapandu HV, Le XF, Mao W, Shentu S (2014). ATP directed agent, 8-chloro-adenosine, induces AMP activated protein kinase activity, leading to autophagic cell death in breast cancer cells. J Hematol Oncol.

