# Supplementary material for: Biological and metabolic effects of IACS-010759, an OxPhos inhibitor, on chronic lymphocytic leukemia cells
Source: Oncotarget. 2018 May 18;9(38):24980–91. doi: 10.18632/oncotarget.25166 (PMC5982765; doi:10.18632/oncotarget.25166)
Supplement: Supplementary file 1 [file oncotarget-09-24980-s001.pdf]

Biological and metabolic effects of IACS-010759, an OxPhos inhibitor, on chronic lymphocytic leukemia cells

SUPPLEMENTARY MATERIALS

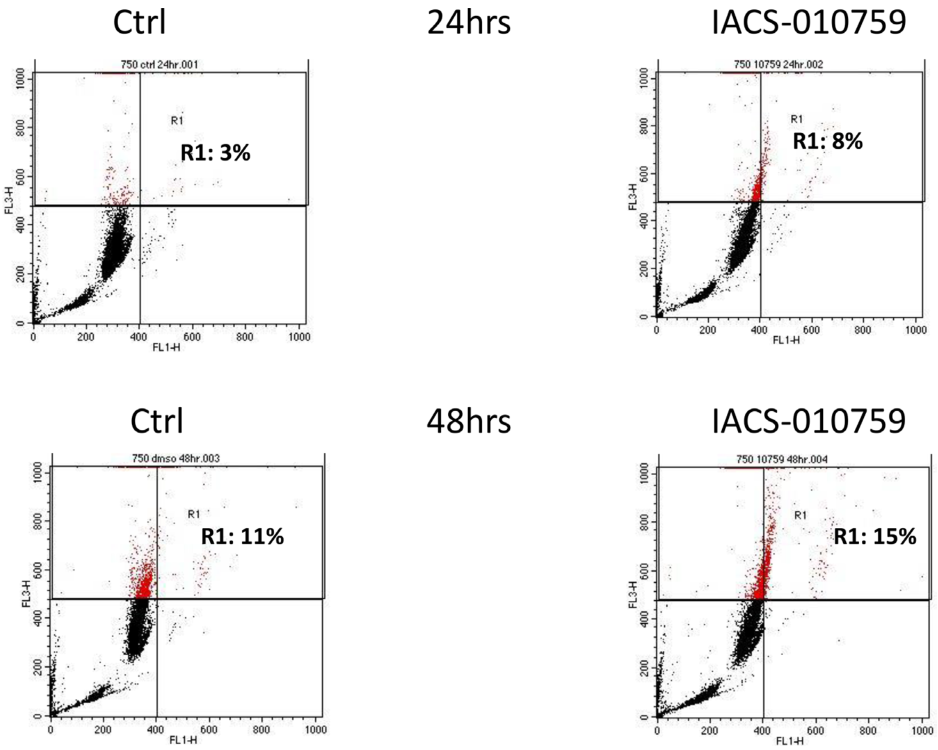

Supplementary Figure 1: Induction of autophagy after treatment with IACS-010759. CLL cells (10^6) from patient #750 were either untreated or treated with IACS-010759 for 24 and 48 h, stained with acridine orange, and analyzed by flow cytometry. R1, % of acridine orange positive CLL cells.

Supplementary Table 1: Patient characteristics. See Supplementary_Table_1
